# Fœtus Papyraceus

**Published:** 1885-08

**Authors:** James H. Etheridge

**Affiliations:** Professor of Materia Medica and Medical Jurisprudence, Rush Medical College, Chicago: Gynecologist to Presbyterian Hospital; Gynecologist to Central Free Dispensary; 65 Randolph Street


THE CHICAGO
### MEDICAL JOURNAL AND EXAMINER.

VOL. LI. AUGUST, 1885. NO. 2.

### ORIGINAL COMMUNICATIONS.

A REPORT OF A CASE OF A FŒTUS ENCLOSED IN ITS SISTER'S PLACENTA. *Fœtus compressus. Fœtus papyraceus. By* JAMES H. ETHERIDGE, A. M., M. D., *Professor of Materia Medica and Medical Jurisprudence, Rush Medical College, Chicago: Gynecologist to Presbyterian Hospital; Gynecologist to Central Free Dispensary.*

(Read before the Chicago Gynecological Society, 16th June, 1885.)

On September 26th, 1882, Mrs. T. J. B., aged twenty-two years, of a nervous sanguine temperament, and healthy, was delivered of a healthy, well-formed female child at term, after a normal labour of four hours' duration. During the delivery of the placenta some abnormity was detectable, which proved to be a *fœtus papyraceus.*

The extreme rarity of such an occurence has led to a careful investigation of the subject, which is herewith recorded.

*The outer surface of the placenta.*—The appearance of the uterine surface of the placentæ at once arrests attention. A deep furrow separates the two placentæ, which are united on their amniotic surfaces by a series of compact white bands discoverable only by pressing through the furrow. The larger placenta constitutes about two-thirds of the entire mass. The smaller placenta is thin, flat and compact, being about one-third as thick as the larger one.

The placenta of the living child is normal throughout its extent. Its cotyledons are well marked, the tufts and villi presenting normal microscopical appearance. It is free from fatty and calcareous degenerations.

The placenta of the *fœtus compressus* in about nine-tenths of its extent is whitish-yellow, very firm, free from cleavage, and it feels much firmer and more compact than the spongy cotyledons of an ordinary placenta.

The whole thickness of this portion of the placenta, excepting its amniotic surface, presents one unbroken mass of fatty degeneration. The remaining one-tenth of the placenta presents a carneous appearance, evidently a transition stage between normal placenta and complete fatty degeneration. Its cotyledons are enmassed, and its tufts and villi solidified, and the whole is interspersed with initial fatty depositions.

*The fœtal surface of the placenta.*

The two segments were wholly different at the time of birth. The placenta of the living child presented an entirely normal appearance. Its cord is inserted about four cm. from the free border away from that of the dead fœtus. The placenta of the *fœtus papyraceus* presented the appearance of a closed bladder, which upon examination was found to be an unruptured amnion containing amniotic fluid and a fœtus. The fœtus, felt at the placenta's delivery, is what constituted the abnormity thus noticed.

*The fœtus.*—The sack, being opened, revealed a compressed fœtus of about three months' growth. It rested with its back towards the free border of the placenta and the anterior surface of the body towards the line of union of the two placentæ.

Its right arm lay in *front* of the chest, the fingers resting near the left acromion process. Its left arm lay in a similar position *behind* the thorax, the fingers resting over the right scapula. The legs were well drawn up till the feet rested on a level with head.

The head was turned so that the chin covered the left shoulder.

In this position the entire fœtus was compressed tightly by its rapidly growing sister till, at birth, it presented the flattened-out appearance which gives it its name, *fœtus compressus*, or *fœtus papyraceus*. The body was flattened from before backward, while the head was flattened from side to side. The two parietal bones made a wedge. The occipital bone lay across the lower segment of the wedge. The two frontal bones made a wedge. The lower jaw also made a wedge. The outline of the head was rhomboidal. The fissures for the eyes were clearly seen. The nose was much flattened. The tongue was especially easily shown.

The cord of the *fœtus papyraceus* was longer by ten centimeters than its fellow, and much thinner. It was inserted quite at the line of union of the two placentæ. It was not unusually twisted.

The following measurements of the fœtus and placenta have been taken:

FŒTUS.

| | | |
|---|---|---|
| Vertex to coccyx........................ | 12 | centimetres. |
| Right arm, including hand........... | 6 | " |
| Left " " " ........... | 7 | " |
| Left thigh.................................... | 3 | " |
| " leg, including foot.................. | 7 | " |
| Acromion to acromion...... .......... | 4½ | " |

Head—

| | |
|---|---|
| Oblique diameter—largest............ | 4 centimetres. |
| " " —smallest........... | 3½ " |
| Straight "............................ | 3½ " |
| Width........................................ | 1⅓ " |

PLACENTA.

1. Of the living child—

| | |
|---|---|
| Long diameter............................ | 12 centimetres. |
| Short " ............................ | 10 " |
| Cord—length.............................. | 30 " |
| " —thickness......................... | 1¼ " |

2. Of the *fœtus papyraceus*—

| | |
|---|---|
| Long diameter............................. | 11 centimetres. |
| Short........................................ | 7 " |
| Amnion.................................... | 9 " |
| Cord—length............................ | 40 " |
| " —thickness........................ | 4-10 " |

PATHOLOGY.

Various causes produce the death of the fœtus. The following may be mentioned:

1st. Faulty insertion of the cord at the margin of the placenta adjoining its fellow, leading to its fatal compression, has been advanced as a possible cause of death of the fœtus. KIESELHAUSEN mentions this faulty cord insertion combined with an extravasation of blood between the placenta and uterus at the location of the placenta of the smaller ovum as the cause of death in the two cases cited in his report (*Dissertatio inauguralis. De fœtibus immaturis uno partu cum maturis editis.*)

2nd. Faulty structure of the cord is another cause of death of the fœtus, as a membranous cord, a thin cord, a twisted cord, or one that is deficient in gelatine, and hence is emaciated and constricted in place.

C. BRAUN found in one case that the cause of death was the membranous insertion of the cord together with its numerous windings about the neck and extremities of the fœtus. (*Breslau. Monatschrift. für Geburtsk. und Frauenkrankh.*, *xiii*, *470*.)

3rd. Disease of the placenta was at one time also given as a cause of death of the fœtus. However, HECKER, in 1861 (*Klinik der Geburtskunde*, *Leipzig*,) and KLEINMEISTER, in 1871 (*Lehre von den Zwillingen*, *Prag*,) considered placental diseases as secondary in the cause of death. It would appear rational to conclude that any systemic condition, as syphilis, would lead to disease of both placentæ instead of but one placenta. Under the head of "*Fibrous obliteration of the placental villi with or without fatty degeneration*" have been described, *induration* of the placenta, *encephaloid placenta*, *scirrhous* placenta, *tuberculous placenta and fatty* placenta. Any one of these conditions may cause the death of the fœtus.

4th. Violence may be reckoned as another cause of death of the fœtus.

I delivered a stout English woman of twins, with *placenta prævia*, where one fœtus was at full term and the other was about four months advanced toward maturity. She affirmed that at that time she fell, one day, against a tub, striking its edge with her abdomen. A slight hæmorrhage occured at the time. Ever after that she felt sore at the point struck. The inference is that a sufficient violence was done to cause a hemorrhage into the villi of the chorion, which caused the death of the fœtus.

5th. Another cause of death of one fœtus may be the implanting too closely together of the umbilical vessels, whence result arterial anastamoses. Thus the blood current of one system may become more powerful than the other, and the main stream becomes diverted toward the more powerful one. The circulation of one fœtus is progressively impaired till it perishes, when at the same time its *liquor amnii* is secreted no more.

*References :*

The extreme rarity of *fœtus papyraceus* is shown in its seldom being reported in current medical literature and in its rarely being mentioned in works on midwifery. A search through the Library of the Surgeon General's office at Washington, resulted in finding five references to reports of similar cases, which are herewith given. It is doubtless true that many cases would be reported were it not for the hasty and imperfect examinations of placentæ made by physicians, which result in completely overlooking the presence of an enclosed fœtus.

The writer has looked through full files of the following named journals, to secure as many references as possible : *The American Journal of the Medical Sciences; The Boston Medical and Surgical Journal ; The New York Medical Record ; The Ohio Medical and Surgical Journal ; The New Orleans Medical and Surgical Journal ; The London Lancet ; The Times and Gazette, London* (*9 years*) ; *The American Journal of Obstetrics and Diseases of Women and Children ; The Obstetrical Journal of Great Britain and Ireland; Annales de Gynécologie ; Archives des Tocologie des medicals des femmes, etc. ; The British Medical Journal* (*12 years.*). All of the "*Text Books of Medicine and Surgery*" *of the New Sydenham Society. Braithwaite's Retrospect* (*44 years.*)

In addition to the foregoing, every obtainable systematic treatise on obstetrics has been consulted. Out of all this mass of literature the following references only to cases and to the subject could be culled.

1. Bard's Midwifery, 4th ed., p. 202, N. Y., 1817, contains an excerpt from "*Perfect's Cases,*" v. ii., p. 159, describing an "undeveloped twin fœtus of the size of the little finger."

2. A case reported in the Medical Repository of N. Y., 1813, i. n. s., p. 383.

3. A case reported in the Boston Medical and Surgical Journal, 1854, xlix, p. 163.

4. Dr. C. McGibbon reported a case of fœtus papyraceus in the New Orleans Medical and Surgical Journal, Sept., 1850, which grew till the 4th month and was retained till full term. *Fœtus papyraceus* born first. Other full-grown but still-born.

5. Dr. J. B. Davis, of Franklin Co., Ohio, reported a case in the Ohio Medical and Surgical Journal, Sept., 1850, where the large twin was a seven months fœtus and the *fœtus papyraceus* was about a ten weeks' product, "fresh and free from all signs of putrefaction."

6. Case in the British Medical Journal, 1869, v. i., p. 141.

7. Case reported in the Detroit Lancet, v. iii., p. 204.

8. Case given in the Chicago Medical Journal and Examiner, 1877, v. xxxiv., p. 410.

9. Case reported in the Lyon Medical, 1873, v. xiii., pp. 304–307.

10. Case reported by Paul Ruge in Beiträge zur Geburtshülfe und Gynäkologie, 1870–1872, b. i., p. 141–144.

11. Case reported in same journal, 1872–1873, ii. b., p. 94.

12. Schuster (W) Die Entstehung des Fœtus Papyraceus und sein Vorkommen bei einfachem und doppeltem Chorion. 80 Strassburg, 1876.

13. Dr. Edis exhibited a case of arrested development of fœtus in twins to Lond. Obst. Soc., Oct 3rd, 1883. One fœtus born alive at seven months, and other, expelled seven hours before, was shriveled and its placenta atrophied, having apparently died two months before.—*Brit. Med. Jour.*, v. ii., 1883, p. 778.

14. Dr. J. R. Kirkpatrick exhibited a mummified fœtus of a twin pregnancy, gone to full term, to the Obst. Sect. of the Acad. of Med. of Ireland, Dec. 22, 1882. She had died about the sixth month. There was a single placenta and double membranous sac. "The portion of placenta which belonged to the mummified fœtus was shrunken and degenerated." —*Brit. Med Jour.*, v. i., 1883, p. 210.

15. Case reported by Dr. E. W. Kneppen, of Ligonier, Ind., in the *American Journal of Obstetrics*, v. xvii, 1884.

16. Case reported in the *Boston Medical and Surgical Journal*, Dr. R. Kingman, 1885. v. cxii, p. 12.

17. In Mauriceau's "observations sur la grossesse et l'accouchment des femmes," page 503, (4th edition, Paris, 1740), is given a case which the author attended, October 12th, 1693.

18. In "Burton's Midwifery" (London, 1751), two cases are cited, with the particulars of each are described. In one case the fœtus is described as being "compressed quite flat," and the author even presents a drawing of this fœtus papyraceus. In this rare work is quoted a case from *Johannes Daniel Geyerus*, who found in a full-term placenta "inclusum alterum filiolum ad spithamæ longitudinem."

19. A case is mentioned by Ed. Chapman, ("A Treatise on the Improvement of Midwifery," p. 66, London, 1735,) which is referred to as "an emaciated child."

20. In Smellie's Midwifery, 4th edition, London, 1768, are references to two cases of included fœtus reported to the Academy of Sciences at Paris in the years 1702 and 1729 respectively. In this book is quoted, from Ruysch of Amsterdam (in Tom. i. oburo. 14,) the case of a surgeon's wife, delivered at term of a small fœtus, 6 hours after the birth of a strong live child. The funis of the embryo "was full of hydatids."

21. Denman, in his "Practice of Midwifery," p. 555, (N.Y., 1821,) says that a twin dying in utero "may be quite flattened or compressed into any other form" by the surviving one, and that at the last it "may be detained several days or weeks" after the expulsion of the living child.

22. Joulin, in "Traite Complet d'Accouchements," p. 757, (Paris, 1867,) quotes a case cited by Guilleman, "where the dead fœtus remained two years in the uterus after the delivery of the other twin."

(The last six cases I am able to report through the courtesy of Dr. John Bartlett, whose rare collection of old treatises on midwifery was kindly placed at my disposal.)

65 Randolph Street.

